# Total hip replacement in the congenitally dislocated hip using the Paavilainen technique: 19 hips followed for 1.5–10 years

**DOI:** 10.3109/17453670902876789

**Published:** 2009-06-01

**Authors:** Bjørn Thorup, Inger Mechlenburg, Kjeld Søballe

**Affiliations:** Department of Orthopaedics, Aarhus University HospitalAarhusDenmark

## Abstract

**Background and purpose** Total hip replacement (THR) in congenitally dislocated hips (CDHs) according to Paavilainen includes placement of the cup in the original acetabulum and an extended trochanteric osteotomy with distal advancement of the trochanter. There have only been a few reports describing the outcome of this technique. Thus, we report the results of 19 THRs using the Paavilainen technique.

**Methods** 10 women and 5 men with an average age of 38 (16–73) years at the time of surgery (19 hips) were followed for mean 4.8 (1.5–10) years. The patients were evaluated clinically with the Harris hip score (HHS) and radiographically using the Gruen and Charnley classification.

**Results** All patients experienced substantial improvement in walking ability and relief of pain. Trendelenburg test was positive in 18 hips preoperatively, and only in 1 postoperatively. 1 case had transient incomplete peroneal palsy. There were 4 cases of intraoperative fissures of the proximal femur. No infections occurred, and no aseptic loosening was observed. 3 hips dislocated in the follow-up period; 2 were reduced open and 1 had a closed reduction. Due to wear of the polyethylene, 3 patients needed replacement of the liner.

**Interpretation** These intermediate to long-term results indicate that the Paavilainen technique provides a functional hip with a limited rate of complications. Wear of the polyethylene liner is, however, still an unresolved issue.

## Introduction

Total hip replacement (THR) for correction of the congenitally dislocated hip (CDH) is a technical challenge and different approaches have been used (Masonis et al. 2003, Gul and Masterson 2005, Park et al. 2007). At our department we use the cementless THR procedure described by Paavilainen et al. (1990), whereby the cup is placed at the level of the true acetabulum and a shortening osteotomy of the proximal part of the femur is carried out. There have only been a few reports on the results achieved with this technique (Paavilainen et al. 1990, 1993, Carlsson et al. 2003, Eskelinen et al. 2006). We evaluated the clinical and radiographic results in a series of Paavilainen procedures performed at our hospital over a 10-year period.

## Patients and methods

From 1996 to 2006, 19 CDHs (15 patients, 10 women) were operated on by the senior author (KS) using the Paavilainen technique (Table). The indications for surgery were CDH with walking difficulties and hip pain. The average age at the time of surgery was 38 (16–73) years. 7 hips had been operated on previously with a Schanz osteotomy. The mean follow-up time was 4.8 (1.5–10) years.

**Table:** Table. Demographic data and results of clinical and radiographic examinations of 19 CDHs operated on with the Paavilainen technique. 4 patients were operated on bilaterally and are thus specified by letters a and b in the case column

Case no.	Follow-up months	Sex	Age at surgery	Eftekhar class	Formerly operated	Trendelenburg preop./postop.	HHS preop./postop.	Gruen zones	Dislocation postop.	Liner replacement
1-sin	120	F	38	C	+	+/–	35/92	1(4F)	–	–
2a-dx	120	M	16	C	+	+/–	10/72	0	+	+
2b-sin	108	M	17	D	–	+/–	36/99	1(1F)	–	+
3-dx	120	F	17	D	–	+/–	43/88	0	–	+
4-sin	120	F	19	D	+	–/–	63/96	0	–	–
5-dx	48	M	26	D	+	+/+	50/83	0	–	–
6a-dx	30	F	16	D	+	+/–	45/93	0	–	–
6b-sin	24	F	17	D	+	+/–	42/89	0	–	–
7-dx	48	M	73	D	–	+/–	38/99	0	–	–
8-sin	48	F	36	C	–	+/–	63/91	0	–	–
9-sin	48	M	42	D	–	+/–	42/95	2(1,4F)	–	–
10-sin	120	F	43	C	–	+/–	63/49	1(2A)	–	–
11a-sin	24	F	46	D	–	+/–	46/89	0	–	–
11b-dx	18	F	47	D	–	+/–	46/50	0	–	–
12a-dx	24	M	45	D	–	+/–	22/95	0	–	–
12b-sin	18	M	45	D	–	+/–	51/88	0	+	–
13-dx	24	F	58	D	–	+/–	55/93	0	+	–
14-sin	24	F	64	D	–	+/–	42/91	0	–	–
15-sin	18	F	16	D	+	+/–	43/82	0	–	–
Total	1104				7	18/1		5	3	3
Median	58		38				44/86			

All 15 patients were clinically evaluated preoperatively and at the most recent follow-up with the Harris hip score (HHS). The radiographs were classified as either Eftekhar type C (4 hips) or D (15 hips) (Eftekhar 1971, Eftekhar and Stinchfield 1973) and they were assessed for aseptic loosening using the Gruen and Charnley classification.

**Figure 1.**
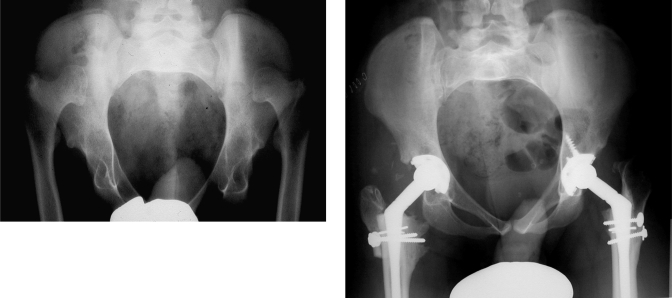
Case no. 2. Congenital dislocated hips bilaterally; grade D according to Efthkhar. A. Preoperatively. B. Postoperatively, the acetabular components (Mallory head) were seated in the true acetabulum. A straight cementless dysplasia stem (Biomet) with a 22-mm head was used. The greater trochanter was fixated with 2 screws. A prophylactic cable was placed around the femur before insertion of the stem.

**Figure 2.**
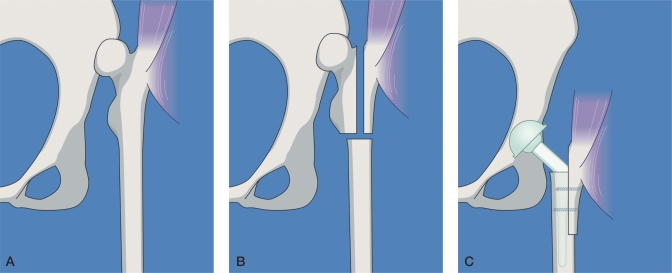
A. CDH grade D. B. Consistent with the preoperative templating, an extended trochanteric osteotomy is performed followed by a transverse osteotomy of the femur. The medial segment is removed. C. A straight stem is inserted and the acetabular component is placed at the level of the true acetabulum. The greater trochanter is advanced distally until the gluteus medius muscle is tight, and then it is fixated with 2 screws.

Preoperatively, templating was performed on radiographs. With the patient standing, the leg-length inequality was estimated by placing wooden blocks of different height (i.e. 0.5, 1, 1.5, 2, 3, 4, and 5 cm) until the position of the pelvis was balanced. In young adults, almost full correction of the leg-length discrepancy was planned whereas in older patients with a more rigid spine, we tried to find the most comfortable lengthening. The operation was performed as described by Paavilainen (1997) using a posterolateral approach (Figures 1 and 2). All acetabular components were seated in the original acetabulum. In most cases, autologous cancellous bone material was placed under the acetabular component before inserting it. We did not need to reinforce the acetabular wall with bone strut grafts in any of the cases. Cementless Mallory-Head cups with hydroxyapatite (HA) coating, 2 or 3 screws, and ArCom polyethylene liner were used in all but 1 hip (case no. 15). This patient had a Duraloc cup (metal-metal articulation).

The femoral shaft was reamed and a trial implant was inserted and trial reduction was performed before insertion of a straight cementless stem. In the first 3 hips, we used Biomet’s dysplasia stem (Biomet, Warsaw, IN) and in the following 16 hips we used S-ROM femoral stems of the dysplastic type (DePuy, Warsaw, IN). After reduction, the greater trochanter was trimmed to fit the lateral part of the proximal diaphysis. With the hip in abduction, the greater trochanter was advanced distally until the gluteus medius muscle was tight, and then fixated with 2 or 3 cortical screws with washers. A cable around the femur was used frequently to avoid fracture of the bone during insertion of the stem. Finally, the vastus lateralis muscle was reinserted at the greater trochanter.

Postoperatively, the patients were mobilized using 2 crutches and allowed 30 kg of weight bearing on the operated leg for the first 6 months. Active abduction of the leg was not allowed in the same period. Full weight bearing was allowed when healing of the osteotomy had been verified radiographically.

## Results (Table)

Preoperatively, the patients had a median HHS of 44; postoperatively, it was 86. The Trendelenburg test was positive in 18 hips preoperatively and in only 1 postoperatively. 1 case of incomplete peroneal nerve palsy (drop foot) occurred and the patient recovered completely after 2 weeks. In 14 of the 15 unilateral hips, the leg length was equalled within 1 cm. In 1 case, the operated leg was 1–2 cm too short. There were 4 cases of intraoperative fissures of the proximal femur. They were all secured with cables and none of these patients experienced postoperative complications. No deep vein thrombosis or early infections were observed.

The radiographic evaluation showed no aseptic loosening of the components. In 3 patients, radiolucency was observed in 1 Gruen zone. In 1 patient, radiolucency was observed in 2 Gruen zones at the time of final follow-up (case no. 9). None of these 4 patients had clinical symptoms of loosening. 3 hips dislocated in the follow-up period; 2 were reduced openly and 1 had a closed reduction. 1 of the dislocations was due to malposition of the acetabular component. The acetabular component was repositioned and no further dislocation occurred. 3 patients needed the liner replaced due to wear of the polyethylene. In 1 of these, deep infection was diagnosed 6 months postoperatively. This patient underwent a 2-stage revision surgery, which cured the infection.

## Discussion

Our good results are in agreement with those of the other 3 follow-up studies on patients with C or D dislocation operated with the same technique (Paavilainen et al. 1993, Carlsson et al. 2003, Eskelinen et al. 2006).

In our series, most of the well-known complications were present but at an acceptable rate. The major problem was the need for reoperation due to wear of the polyethylene liner. Eskelinen et al. (2006) concluded that wear was secondary to suboptimal design of the acetabular components used. We have started using hard bearings in our younger patients: ceramic-ceramic for females and metal-metal for males, which will hopefully be a better solution than polyethylene. Metal bearings may be of concern in women in childbearing age; metal ions pass the placenta (Ziaee et al. 2007). Highly cross-linked polyethylene has shown very low wear rates (Digas et al. 2007), and the use of this instead of conventional polyethylene is also a possible solution in young, active patients.

We experienced 2 dislocations within 2 days of surgery. The first hip was reduced closed without any further complications. The second dislocation was due to malposition of the cup, and an open reduction was performed. In addition, we performed an open reduction on a chronic dislocation discovered at a routine check 3 months after it had occurred. The patient had noted pain and shortening of the leg, but had not found it necessary to contact a doctor. Both Paavilainen et al. (1993) and Carlsson et al. (2003) reported late dislocations in their series, and a plausible reason for the relatively high dislocation rate was the use of small 22-mm heads.

We had 1 case of temporary incomplete peroneal palsy in a patient whose leg had been lengthened 3 cm. The nerve monitoring with electric-evoked potentials during operation did not indicate problems and the palsy disappeared 2 weeks postoperatively. Eggli et al. (1999) found no correlation between lengthening of the leg and nerve palsy and concluded that nerve contusion was responsible in most cases. Eskelinen et al. (2006) reported peroneal palsy in 3 of 68 patients in their series.

One problem that occurred during operation in all series—our own included—was a fissure in the proximal femur. We stabilized the 4 fractures we experienced with Dall-Miles cable, with good results, and used a prophylactic cable in several other patients. A potential complication is nonunion between the greater trochanter and the proximal diaphysis (Sanchez-Sotelo et al. 2002) but we did not encounter this complication.

In conclusion, THR using the Paavilainen technique has provided our series of patients with a stable and functional hip, with a limited rate of complications. We are mainly concerned about the wear of the polyethylene, which is excessive in these mostly younger patients. This is thus a problem that must be considered.
